# Comparison of Methods for Alcohol and Drug Screening in Primary Care Clinics

**DOI:** 10.1001/jamanetworkopen.2021.10721

**Published:** 2021-05-20

**Authors:** Jennifer McNeely, Angéline Adam, John Rotrosen, Sarah E. Wakeman, Timothy E. Wilens, Joseph Kannry, Richard N. Rosenthal, Aimee Wahle, Seth Pitts, Sarah Farkas, Carmen Rosa, Lauren Peccoralo, Eva Waite, Aida Vega, Jennifer Kent, Catherine K. Craven, Tamar A. Kaminski, Elizabeth Firmin, Benjamin Isenberg, Melanie Harris, Andre Kushniruk, Leah Hamilton

**Affiliations:** 1 Department of Population Health, New York University Grossman School of Medicine, New York; 2 Department of Medicine, Division of General Internal Medicine, New York University Grossman School of Medicine, New York; 3 Department of Psychiatry, University Hospital Lausanne, Lausanne, Switzerland; 4 Department of Psychiatry, New York University Grossman School of Medicine, New York; 5 Department of Medicine, Harvard Medical School, Massachusetts General Hospital, Boston; 6 Department of Psychiatry, Massachusetts General Hospital, Boston; 7 Division of General Internal Medicine, Icahn School of Medicine at Mount Sinai, New York, New York; 8 Renaissance School of Medicine, Stony Brook University, Stony Brook, New York; 9 The Emmes Company, Rockville, Maryland; 10 National Institute on Drug Abuse, Bethesda, Maryland; 11 Department of Population Health Science and Policy, Icahn School of Medicine at Mount Sinai, New York, New York; 12 School of Health Information Science, University of Victoria, Victoria, British Columbia, Canada

## Abstract

**Importance:**

Guidelines recommend that adult patients receive screening for alcohol and drug use during primary care visits, but the adoption of screening in routine practice remains low. Clinics frequently struggle to choose a screening approach that is best suited to their resources, workflows, and patient populations.

**Objective:**

To evaluate how to best implement electronic health record (EHR)–integrated screening for substance use by comparing commonly used screening methods and examining their association with implementation outcomes.

**Design, Setting, and Participants:**

This article presents the outcomes of phases 3 and 4 of a 4-phase quality improvement, implementation feasibility study in which researchers worked with stakeholders at 6 primary care clinics in 2 large urban academic health care systems to define and implement their optimal screening approach. Site A was located in New York City and comprised 2 clinics, and site B was located in Boston, Massachusetts, and comprised 4 clinics. Clinics initiated screening between January 2017 and October 2018, and 93 114 patients were eligible for screening for alcohol and drug use. Data used in the analysis were collected between January 2017 and October 2019, and analysis was performed from July 13, 2018, to March 23, 2021.

**Interventions:**

Clinics integrated validated screening questions and a brief counseling script into the EHR, with implementation supported by the use of clinical champions (ie, clinicians who advocate for change, motivate others, and use their expertise to facilitate the adoption of an intervention) and the training of clinic staff. Clinics varied in their screening approaches, including the type of visit targeted for screening (any visit vs annual examinations only), the mode of administration (staff-administered vs self-administered by the patient), and the extent to which they used practice facilitation and EHR usability testing.

**Main Outcomes and Measures:**

Data from the EHRs were extracted quarterly for 12 months to measure implementation outcomes. The primary outcome was screening rate for alcohol and drug use. Secondary outcomes were the prevalence of unhealthy alcohol and drug use detected via screening, and clinician adoption of a brief counseling script.

**Results:**

Patients of the 6 clinics had a mean (SD) age ranging from 48.9 (17.3) years at clinic B2 to 59.1 (16.7) years at clinic B3, were predominantly female (52.4% at clinic A1 to 64.6% at clinic A2), and were English speaking. Racial diversity varied by location. Of the 93,114 patients with primary care visits, 71.8% received screening for alcohol use, and 70.5% received screening for drug use. Screening at any visit (implemented at site A) in comparison with screening at annual examinations only (implemented at site B) was associated with higher screening rates for alcohol use (90.3%-94.7% vs 24.2%-72.0%, respectively) and drug use (89.6%-93.9% vs 24.6%-69.8%). The 5 clinics that used a self-administered screening approach had a higher detection rate for moderate- to high-risk alcohol use (14.7%-36.6%) compared with the 1 clinic that used a staff-administered screening approach (1.6%). The detection of moderate- to high-risk drug use was low across all clinics (0.5%-1.0%). Clinics with more robust practice facilitation and EHR usability testing had somewhat greater adoption of the counseling script for patients with moderate-high risk alcohol or drug use (1.4%-12.5% vs 0.1%-1.1%).

**Conclusions and Relevance:**

In this quality improvement study, EHR-integrated screening was feasible to implement in all clinics and unhealthy alcohol use was detected more frequently when self-administered screening was used at any primary care visit. The detection of drug use was low at all clinics, as was clinician adoption of counseling. These findings can be used to inform the decision-making of health care systems that are seeking to implement screening for substance use.

**Trial Registration:**

ClinicalTrials.gov Identifier: NCT02963948

## Key Points

**Question:**

How are commonly used screening methods for alcohol and drug use associated with implementation outcomes among adult patients in primary care clinics, and what is the best approach for implementing electronic health record–integrated screening?

**Findings:**

In this quality improvement study implementing systematic screening for alcohol and drug use among 93 114 patients in 6 primary care clinics, 72% of patients completed screening. Screening at any visit (in comparison with screening at annual examinations only) was associated with higher screening rates for alcohol and drug use, and self-administered screening was associated with greater detection of moderate- to high-risk alcohol use compared with staff-administered screening.

**Meaning:**

These findings suggest that, to maximize the adoption of substance use screening during primary care visits, clinics can conduct screening at any visit and use self-administered screening tools to increase the detection of unhealthy alcohol use among adult patients.

## Teaser

This quality improvement study compares commonly used screening methods for alcohol and drug use among adult patients at primary care clinics to examine their association with implementation outcomes and to evaluate the best approach for implementing electronic health record–integrated screening.

## Introduction

Alcohol and drug use are among the top 10 causes of preventable death in the US.^1^ More than 72 500 deaths in 2017 were associated with alcohol use,^2^ and drug overdose deaths now exceed 81 000 deaths per year.^3^ The US Preventive Services Task Force recommends screening for both alcohol and drug use among adult patients during primary care visits.^4,5^ While the drug screening recommendation reflects a recent change, alcohol screening has been recommended for more than 2 decades and ranks as the third-highest prevention priority for adults in the US.^6,7^

Despite the substantial health burden of alcohol and drug use, screening and interventions to address these issues are rarely incorporated into routine medical care.^8,9,10,11,12^ Barriers to screening for alcohol use include systems-level problems, such as time and workflow constraints, and underlying issues of stigma and lack of clinician knowledge.^13,14,15,16,17^ These barriers are even more pronounced for drug screening because of the variety of substances (ranging from cannabis to heroin and the nonmedical use of prescribed medications), the illegality of some drugs, the greater stigmatization among patients, and the knowledge deficits among clinicians.^18,19,20^

Electronic health records (EHRs), which can facilitate systematic screening, guide clinician actions, and record results in structured data fields, have been underused for substance use.^21,22^ Common data elements for alcohol and drug information have been defined and recommended for integration into EHRs^23^; however, in most systems, this information is still gathered in social history fields that do not include validated screening questionnaires and are inconsistently used.

To inform strategies for implementing substance use screening and interventions in primary care settings, the Clinical Trials Network of the National Institute on Drug Abuse conducted a study of EHR-integrated screening in 2 large urban academic health care systems. The primary goal was to facilitate the implementation of screening approaches that were feasible, had good potential for sustainability, and would optimize the screening rate and capture of screening data in the EHR. To evaluate how best to implement EHR-integrated screening, the present study compared screening methods to examine their association with implementation outcomes during the first year of screening at 6 participating primary care clinics.

## Methods

This study was approved by the institutional review boards of the New York University Grossman School of Medicine, the Icahn School of Medicine at Mount Sinai, and Partners Healthcare System. A waiver of informed consent was granted because the screening was conducted as part of routine clinical care, the study posed minimal risk to participants, and it would not have been feasible to obtain individual consent from all clinic patients. The study followed the Standards for Quality Improvement Reporting Excellence (SQUIRE) reporting guideline for health care quality improvement studies.^24^

### Study Design and Setting

In this quality improvement study assessing implementation feasibility, clinics were required to use screening tools included in the National Institute on Drug Abuse Common Data Elements.^23^ All clinics received support to compensate a clinical champion (10% full-time equivalent) and expert consultation from the research team to assist them in choosing a screening approach that clinic leaders believed would be the most feasible and effective in their setting. The clinics used existing EHR systems and were not provided with additional clinical staff or resources to perform screening. Although the study was not randomized, clinics varied in their screening approaches and implementation strategies. We sought to characterize and evaluate the screening implementation outcomes achieved using these diverse tactics.

The study sites were 2 urban academic health care systems; site A was in New York City, and site B was in Boston, Massachusetts. At site A, which included 2 primary care clinics (A1 and A2), screening was advocated by a site principal investigator (R.N.R.), who is a clinical leader in the treatment of substance use disorders. At site B, which included 4 primary care clinics (B1 through B4), screening was part of a systemwide initiative to integrate substance use care into general medical settings. Clinics were general internal medicine practices serving adult patients, and none of the clinics were systematically screening patients for alcohol or drug use before the study began. One clinic from each site was the primary teaching practice for the internal medicine residency program, which was located near the hospital. The other clinics were in community settings, and 1 clinic (A2) was a faculty practice. All clinics used Epic EHR software (Epic Systems Corp).

Before the initiation of screening, barriers were assessed through focus groups and interviews with stakeholders,^25^ and the EHR tools developed to support screening were tailored through multiple rounds of usability testing. Screening was initiated at 1 clinic within each health care system in January 2017 (clinic A1) and July 2017 (clinic B1). Screening was then implemented at the remaining clinics (A2, B2, B3, and B4) between February and October 2018. Implementation outcomes were collected for 12 months after initiation of screening at each clinic, and data collection ended in October 2019. The primary outcome was the screening rate for alcohol and drug use. Secondary outcomes were the prevalence of unhealthy substance use detected via screening and clinician adoption of a brief counseling script.

### Screening Program Elements

Clinics used screening program elements that had been reported to increase the adoption of screening and interventions by health care professionals.^26,27,28,29^ These elements^23,30,31,32,33,34,35,36,37^ are summarized in Table 1 and included the training of clinic staff and the use of validated screening tools (the single-item screening questions for alcohol and drugs, the 3-item Alcohol Use Disorders Identification Test–Consumption items [AUDIT-C] and the 10-item Drug Abuse Screening Test [DAST-10]), a clinical reminder in the EHR indicating that a patient was due for screening (based on age, type of visit, and no receipt of screening within the past 12 months), a brief EHR-integrated counseling script suggested for use with patients with moderate- to high-risk alcohol or drug use, and clinical champions (ie, clinicians who advocate for change, motivate others, and use their expertise to facilitate the adoption of an intervention). The counseling script was created for the study and included the 4 major components of a brief negotiated interview: raising the subject, providing feedback, enhancing motivation, and negotiating a plan.^36^

**Table 1.** Screening Program Elements Common to All Clinics

Element	Description
EHR	All sites used Epic EHR software
	Site B used a custom-built user interface to administer screening on tablets alongside other patient-reported outcome measures
Screening tools	Validated screening tools that were designated as NIDA common data elements^23^
	All patients received single-item screening questions for alcohol and drug use; responses >0 were considered positive results^30,31,32^
	Patient with positive alcohol screening results received the AUDIT-C^33^; patients with positive drug screening results received the DAST-10,^34,35^ which provides a single summary score and does not specify drug classes used
	Established cutoffs were used to categorize results as representing moderate- or high-risk use^a^
Clinical reminders	Best practice alert appears in the EHR, indicating that a patient is due for screening (based on age, visit type, and no screening within the past 12 mo)
Counseling script	EHR-integrated counseling script created for the study provided guidance for conducting and documenting a brief intervention to address substance use
	Training of clinic staff recommended using the script for patients with moderate- to high-risk alcohol or drug use
	Accessed through a dot phrase in Epic EHR software: 1 keystroke to start, with fillable fields to document patient responses (2 fields at site A and up to 7 fields at site B)
	Designed to be delivered in approximately 5 min
	Guided clinicians through the 4 major components of a brief negotiated interview: raising the subject, providing feedback, enhancing motivation, and negotiating a plan^36^
	For patients with high-risk use, the script suggested placing a referral order for an appointment with clinic social workers or a peer navigator
Clinical champions	Each clinic had 1 or 2 designated clinical champions
	Champions worked with research team and led implementation at their clinics
	Met approximately once per month with the research staff
	Small amount of support provided by the study (total of 10% FTE per clinic allocated to 1 or 2 champions)
Training	Conducted by research staff
	Offered during established meeting times to facilitate attendance
	Clinicians: 1 group training session on screening, brief intervention, and use of EHR tools (30-45 min)
	Medical residents: 1-h educational session at beginning of ambulatory care rotations
	Medical assistants and front desk staff: 1 brief training focused on screening workflow^b^
	Clinicians and medical assistants who were unable to attend group training could receive individual training

Abbreviations: AUDIT-C, Alcohol Use Disorders Identification Test–Consumption items; DAST-10, 10-item Drug Abuse Screening Test; EHR, electronic health record; FTE, full-time equivalent; NIDA, National Institute on Drug Abuse.

^a^ Cutoff scores used for the AUDIT-C: for moderate-risk alcohol use, 3 to 7 points for women and 4 to 7 points for men; for high-risk alcohol use, 8 points or higher. Cutoff scores used for the DAST-10: for moderate-risk drug use, 3 to 5 points; for high-risk drug use, 6 points or higher.^33,34,35,37,38^

^b^ Medical assistants at clinic A1 received 3 additional training sessions in verbal administration of screening.

### Screening Approach and Implementation Strategies

Participating health care systems and clinics selected the screening approach that they deemed most feasible given their resources, clinical workflows, and patient populations. There was variation in the type of visit targeted for screening (annual examination only vs any visit), with clinics A1 and A2 choosing to conduct screenings at any visit and clinics B1 through B4 choosing to conduct screenings at annual examinations only (Table 2). The mode of screening administration (staff-administered vs self-administered by the patient) also varied, with 5 clinics choosing self-administration and only 1 clinic (A1) choosing staff administration (Table 2).

**Table 2.** Screening Approaches and Implementation Strategies Used at Each Clinic

Clinic	Screening approach		Implementation strategy	
	Visit type targeted for screening^a^	Mode of screening administration^b^	Practice facilitation^c^	Individuals participating in usability testing, No.^d^
A1	Any	Staff-administered	Robust	18
A2	Any	Self-administered	Robust	9
B1	Annual examination	Self-administered	Standard	5
B2	Annual examination	Self-administered	Standard	4
B3	Annual examination	Self-administered	Standard	7
B4	Annual examination	Self-administered	Standard	0

Abbreviations: AUDIT-C, Alcohol Use Disorders Identification Test–Consumption items; DAST-10, 10-item Drug Abuse Screening Test.

^a^ At site A clinics, screening was administered at any in-person primary care visit. At site B clinics, only annual preventive care or physical examination visits were targeted for screening.

^b^ For staff-administered screening, a medical assistant completed the single-item screening instrument and, if positive, the primary care clinician completed the AUDIT-C or DAST-10. Self-administered screening was typically completed on a tablet or kiosk in the waiting room. At clinic A2, patients also had the option of completing screening on the patient portal before their visit.

^c^ Robust practice facilitation was distinguished by a high frequency (1-3 d/wk) of on-site involvement by a study research coordinator who had specific training in practice facilitation and regular monthly meetings with the clinical champions to review implementation data reports. Standard practice facilitation was undertaken by research coordinators who did not receive specific facilitation training, were on site less than 1 d/wk, and had ad hoc meetings with clinical champions.

^d^ Usability testing was performed by primary care clinicians at all clinics. At clinic A1, additional testing was performed by 3 medical assistants.

Clinics varied in the extent of practice facilitation and usability testing of the EHR tools developed to support screening. Practice facilitation is an implementation strategy in which trained facilitators work with clinic leaders and staff to implement evidence-based practices.^39,40,41^ All clinics received practice facilitation from study investigators (T.A.K., E.F., B.I., and M.H.) for the first 12 months of the screening program, and the extent of facilitation was classified as robust vs standard based on training, days spent on site by the facilitator, and frequency of meetings with clinical champions. Usability testing involves observing human-computer interaction as representative end users use a prototype and provide feedback on system design, interface, information content, and mode of delivery. It is an iterative process in which each round of usability testing is followed by system redesign based on user feedback.^42,43^ Three rounds of usability testing were conducted at clinic A1 (performed by J.M., J.K., M.H., and A.K.), 2 rounds were conducted at clinic A2 (performed by J.M., J.K., C.K.C., M.H., and A.K.), and 1 round was conducted at clinics B1 through B4 (performed by T.A.K. and B.I.). At site B, substance use screening was incorporated into a larger primary care initiative to collect patient-reported information on a broad range of topics; in comparison with site A, site B had less flexibility to adapt the EHR and less time to allocate to practice facilitation.

### Screening Implementation Outcomes

Implementation outcome data were extracted from the EHR for 12 months after initiation of the screening program at each clinic. Data collected comprised (1) the number of patients eligible for screening (adults aged ≥18 years who had ≥1 primary care visit and had not completed screening in the preceding 12 months), (2) the proportion of eligible patients screened for alcohol and/or drug use, (3) the prevalence and risk level of unhealthy alcohol and drug use detected on screening, and (4) clinician adoption of the suggested counseling script for patients who had positive screening results for moderate- to high-risk alcohol and/or drug use, as detected by an EHR data element embedded in the counseling script. At each clinic, summary reports of implementation outcomes were generated weekly for the first 3 months of screening and quarterly thereafter. Reports were sent to the investigators and research staff, who then shared and discussed them with clinical champions.

### Statistical Analysis

Clinics sent extracted EHR data that captured screening information to the study’s data and statistical coordinating center (The Emmes Company, Rockville, Maryland) throughout the first 12 months; these data were used to generate summary reports of the implementation outcome measures for each clinic. A separate data extraction was performed at the end of the study period to collect sociodemographic information from the EHR, which was used to describe the patient populations at each clinic. Descriptive statistics were used to characterize the rates and frequencies of predefined implementation outcomes.

Screening rate was calculated as the proportion of eligible patients presenting for a primary care visit who completed screening for alcohol or drug use. Patients who had a positive result on the single-item screening question for alcohol (“How many times in the past year have you had *x* or more drinks in a day?” [*x* was 5 for men and 4 for women])^30,31^ received the AUDIT-C (score range, 0-12 points, with higher scores indicating a greater likelihood of having an unhealthy level of alcohol use or an alcohol use disorder). Patients who had a positive result on the single-item screening question for drugs (“How many times in the past year have you used an illegal drug or used a prescription medication for nonmedical reasons [for example, because of the experience or feeling it caused]?”)^30,32^ received the DAST-10 (score range, 0-10 points, with higher scores indicating more severe levels of problems associated with drug use).^32,34,35,37^ A positive result for the single-item screening questions was defined as any response greater than 0 points. The prevalence of moderate- to high-risk use was calculated using standard cutoffs for the AUDIT-C (for moderate-risk alcohol use, 3-7 points for women and 4-7 points for men; for high-risk alcohol use, ≥8 points) and the DAST-10 (for moderate-risk drug use, 3-5 points; for high-risk drug use, ≥6 points)^33,34,35,37,38^ (Table 1). Because the counseling script was only suggested for use among patients who received positive screening results for moderate- to high-risk use of alcohol and/or drugs, the counseling rate was calculated based on the moderate- to high-risk population. Data used in the analysis were collected between January 2017 and October 2019, and analysis was performed from July 13, 2018, to March 23, 2021, using SAS software version 9.4 (SAS Institute Inc).

## Results

Patients of the 6 clinics had a mean (SD) age ranging from 48.9 (17.3) years at clinic B2 to 59.1 (16.7) years at clinic B3, were predominantly female (52.4% at clinic A1 to 64.6% at clinic A2), and were English speaking. Racial diversity varied by location. Other characteristics of the participating clinics and their patient populations are shown in Table 3. Of the 6 clinics included in the study, the approximate number of patient visits per year ranged from 12 000 at clinic B4 to 60 000 at clinic A1; clinic B3 had the highest number of attending physicians (50), and clinic A1 had the highest number of medical residents (134). The site B clinics, with the exception of clinic B2, served a predominantly White patient population, while site A clinics had a higher proportion of patients who were Black or of other races (a category that included patients who identified as having Hispanic ethnicity without specifying their race). Insurance status varied by practice type, with clinic A1 having the largest proportion of Medicaid patients (41.8%), clinic B3 having the largest proportion of Medicare patients (37.0%), and clinic A2 (the faculty practice) having the highest proportion of privately insured patients (81.0%).

**Table 3.** Characteristics of Participating Clinics and Patient Populations

Characteristic	No. (%)					
	Clinic A1	Clinic A2	Clinic B1	Clinic B2	Clinic B3	Clinic B4
Clinics						
Approximate patient visits per year, No.	60 000	50 000	13 000	21 000	37 000	12 000
Attending physicians, No.	20	18	13	20	50	11
Medical residents, No.^a^	134	0	10	10	64	4
Patients^b^						
Age, mean (SD), y	55.6 (17.2)	50.0 (17.1)	51.6 (16.4)	48.9 (17.3)	59.1 (16.7)	53.7 (17.8)
Sex^c^						
Male	6152 (35.4)	9549 (37.3)	3215 (45.2)	4207 (38.5)	12 036 (47.6)	2573 (38.3)
Female	11 210 (64.6)	16 085 (62.7)	3892 (54.8)	6707 (61.5)	13 250 (52.4)	4147 (61.7)
Race						
Black or African American	4931 (28.4)	3694 (14.4)	350 (4.9)	843 (7.7)	1478 (5.8)	278 (4.1)
White	2911 (16.8)	10 358 (40.4)	5882 (82.8)	3904 (35.8)	20 593 (81.4)	4627 (68.9)
Asian	615 (3.5)	1909 (7.4)	250 (3.5)	302 (2.8)	1536 (6.1)	789 (11.7)
Other^d^	8905 (51.3)	9676 (37.7)	625 (8.8)	5866 (53.7)	1679 (6.6)	1026 (15.3)
Health insurance						
Medicaid	7254 (41.8)	24 (0.1)	1169 (16.4)	3871 (35.5)	1255 (5.0)	1405 (20.9)
Medicare	5325 (30.7)	4665 (18.2)	1870 (26.3)	2444 (22.4)	9356 (37.0)	2089 (31.1)
Private	4448 (25.6)	20 773 (81.0)	4031 (56.7)	4229 (38.7)	14 616 (57.8)	3102 (46.2)
None	3 (0.02)	3 (0.01)	0	0	0	0
Other^e^	9 (0.05)	77 (0.3)	37 (0.5)	371 (3.4)	59 (0.2)	124 (1.8)
Missing	323 (1.9)	95 (0.4)	0	0	0	0
Language						
English	15 051 (86.7)	25 052 (97.7)	6706 (94.4)	5983 (54.8)	23 893 (94.5)	5690 (84.7)
Spanish	1987 (11.4)	394 (1.5)	235 (3.3)	4062 (37.2)	409 (1.6)	352 (5.2)
Other	228 (1.3)	99 (0.4)	119 (1.7)	845 (7.7)	817 (3.2)	660 (9.8)
Missing	96 (0.6)	92 (0.4)	47 (0.7)	25 (0.2)	167 (0.7)	18 (0.3)

^a^ Clinics A1 and B3 were the primary outpatient clinical training sites for residents in general medicine at their respective sites.

^b^ Characteristics of adult patients who had primary care visits during the study period. Demographic data were extracted from the electronic health record separately from implementation outcome data; therefore, there are small discrepancies in the number of patients included in Table 3 (N = 93 023) and the number included in the implementation outcomes data shown in Table 4 (N = 93 114).

^c^ At clinic A2, 3 patients reported their sex as other. At clinic B2, 1 patient reported their sex as other.

^d^ Other races included American Indian/Alaska Native, Native Hawaiian/other Pacific Islander, other, multiracial, unavailable or declined, and missing. Hispanic ethnicity was an optional field in the EHRs and was missing for most patients; therefore, it was not included as an additional variable in the study.

^e^ Other insurance included coverage from the US Department of Veterans Affairs, workers’ compensation, professional associations (eg, law enforcement), county jails, Massachusetts Health Safety Net, and hospital pay.

Among 93 114 patients eligible for screening, 66 817 patients (71.8%) received screening for alcohol use, and 65 656 patients (70.5%) received screening for drug use (Table 4). Screening rates were higher among clinics that conducted screening at any visit (for alcohol screening, 90.3% at clinic A1 and 94.7% at clinic A2; for drug screening, 89.6% at clinic A1 and 93.9% at clinic A2) compared with clinics that conducted screening at annual examinations only (for alcohol screening, 42.2% at clinic B1, 24.2% at clinic B2, 72.0% at clinic B3, and 44.3% at clinic B4; for drug screening, 37.9% at clinic B1, 24.6% at clinic B2, 69.8% at clinic B3, and 44.1% at clinic B4) (Table 4 and eFigure in the Supplement).

**Table 4.** Implementation Outcomes for First 12 Months of Screening

Outcome	No./total No. (%)					
	Clinic A1	Clinic A2	Clinic B1	Clinic B2	Clinic B3	Clinic B4
Total patients, No.^a^	17 373	25 632	7139	10 932	25 311	6727
Received screening for alcohol	15 687/17 373 (90.3)	24 270 /25 632 (94.7)	3016/7139 (42.2)	2648/10 932 (24.2)	18 214/25 311 (72.0)	2982/6727 (44.3)
Receiving screening for drugs	15 558/17 373 (89.6)	24 064/25 632 (93.9)	2708/7139 (37.9)	2689/10 932 (24.6)	17 670/25 311 (69.8)	2967/6727 (44.1)
Risk level for alcohol use^b^						
Moderate-high	253/15 687 (1.6)	3562/24 270 (14.7)	1105/3016 (36.6)	513/10 932 (19.4)	6179/18 214 (33.9)	638/2982 (21.4)
Moderate	194/15 687 (1.2)	3420/24 270 (14.1)	1041/3016 (34.5)	480/10 932 (18.1)	6047/18 214 (33.2)	613/2982 (20.6)
High	59/15 687 (0.4)	142/24 270 (0.6)	64/3016 (2.1)	33/10 932 (1.3)	132/18 214 (0.7)	25/2982 (0.8)
Risk level for drug use^b^						
Moderate-high	78/15 558 (0.5)	64/24 064 (0.3)	28/2708 (1.0)	28/10 932 (1.0)	70/17 670 (0.4)	25/2967 (0.8)
Moderate	59/15 558 (0.4)	59/24 064 (0.2)	11/2708 (0.4)	20/10 932 (0.7)	48/17 670 (0.3)	15/2967 (0.5)
High	19/15 558 (0.1)	5/24 064 (0.02)	17/2708 (0.6)	8/10 932 (0.3)	22/17 670 (0.1)	10/2967 (0.3)
Received counseling^c^	39/311 (12.5)	49/3587 (1.4)	2/1129 (0.2)	6/533 (1.1)	6/6233 (0.1)	3/655 (0.5)

^a^ Total patients eligible for screening were adults who attended at least 1 visit with a primary care clinician. Urgent care visits were excluded.

^b^ Calculated as a proportion of patients screened.

^c^ Counseling was tracked through a data element included in the suggested counseling script. Counseling rate was measured as percentage of those with screening results indicating moderate- or high-risk alcohol or drug use, although the counseling script could be used with any patient.

The prevalence of moderate- to high-risk alcohol use detected by screening was lowest at clinic A1 (1.6%), which used a staff-administered screening approach. At clinics that used a self-administered approach, the prevalence of moderate- to high-risk alcohol use was higher, ranging from 14.7% at clinic A2 to 36.6% at clinic B1. The prevalence of moderate- to high-risk drug use detected by screening was low at all clinics, ranging from 0.3% at clinic A2 to 1.0% at clinics B1 and B2.

The counseling script was used infrequently, with rates ranging from 0.1% at clinic B3 to 12.5% at clinic A1 (Table 4). Clinics A1 and A2, which used implementation strategies comprising robust practice facilitation and more EHR usability testing, had the highest rates of counseling (12.5% and 1.4%, respectively) across the 6 clinics.

## Discussion

In this quality improvement study of the feasibility and implementation of EHR-integrated screening, 71.8% of eligible patients received screening for substance use over the course of 12 months, via the use of validated questionnaires during routine primary care visits. This screening represents a substantial change in practice for the participating clinics, none of which were systematically screening patients for alcohol or drug use before our study intervention began. The successful implementation of screening aligns these clinics with the current US Preventive Services Task Force guidelines for alcohol and drug screening among adult patients in primary care settings.^4,5^

Notably, the screening program was implemented using existing clinic staff and EHRs, although the study provided support for practice facilitation and usability testing as well as modest funding for clinical champions. Screening rates were higher than those observed in a number of previous screening implementation studies.^26,29,44^ At some clinics, screening prevalence was consistent with results from the Veterans Health Administration, which has prioritized alcohol screening during primary care visits for years.^45^ However, we did observe differences in screening rates that appeared to be associated with the screening approach and implementation strategies used in the participating clinics.

Screening rates were highest at site A clinics, which adopted the approach of screening patients during any primary care visit. The screening rates at site A (89.6%-94.7%) were slightly higher than those reported in a recent pragmatic clinical trial conducted at primary care practices in Washington state.^46^ The site B clinics, which specifically targeted annual examinations for screening, had lower and more variable screening rates (24.2%-72.0%). Although it is typical for primary care practices to conduct screening only during a dedicated annual physical or preventive care visit, patients may miss appointments; therefore, offering screening during any type of visit provides more opportunities to detect substance use. Notably, at all sites, the performance of screening was suggested only once per year because screening at every visit may produce patient and clinician fatigue and decrease the accuracy of results.^47^

Perhaps the most notable finding was the substantially lower detection of unhealthy alcohol use at the 1 clinic (A1) that used a staff-administered screening approach in comparison with clinics that used a self-administered approach. Based on survey data, the anticipated prevalence of unhealthy alcohol use in the general population is approximately 30%.^48,49^ In medical settings, the rates of positive screening results are typically lower, which may be associated with patients’ reluctance to disclose substance use.^25,50,51,52^ Two study clinics achieved rates of positive screening results for moderate- to high-risk alcohol use that exceeded general population estimates, and all 5 clinics using a self-administered approach detected a greater than 14% prevalence of moderate- to high-risk alcohol use (vs <2% for the clinic that used staff-administered screening). Our findings are consistent with those of previous studies using self-administered screening, which typically produces more accurate reporting among patients with stigmatized conditions.^53,54,55^ Our findings could also reflect problems with the quality of screening when administered by staff, who may change the wording of validated screening questions in an effort to hasten the process or reduce perceived patient discomfort.^56,57^

Screening rates were similar for alcohol and drug use, likely reflecting the fact that most clinics administered the alcohol and drug screenings simultaneously. However, the detection of unhealthy drug use via screening was low and did not appear to vary based on the screening approach or implementation strategy used. Although population rates of illicit drug use are lower than those of alcohol use, the prevalence of past-year drug use may be as high as 21% when cannabis is included.^58^ The low screening-detected prevalence of drug use likely reflects patients’ discomfort with disclosing a behavior that is illegal and stigmatized and one that they may believe could negatively impact their medical care. The drug screening tools used in our study did not distinguish between the use of cannabis and other drugs. Given the changing legal status and social acceptability of cannabis, it is possible that using a screening instrument that contains separate questions about cannabis and other illicit drugs, such as the Tobacco, Alcohol, Prescription Medications, and Other Substance Use (TAPS) tool,^59^ would produce higher reporting of drug use.^60,61^ Regardless of the screening approach, increasing patients’ comfort with disclosing drug use may also require improving clinicians’ attitudes.^17,19,25^

Clinics differed in their implementation strategies, with site A clinics having more robust practice facilitation and usability testing than site B clinics, which may have been associated with the higher rates of adoption of screening and counseling. Practice facilitation can be a beneficial implementation strategy, although results are not uniform across studies.^39,40,41^ Usability testing is helpful for implementing any practice change that involves modifications to the EHR.^43,62,63^ In our study, it is not possible to differentiate the relative contributions of practice facilitation and usability testing, although the clinic that performed the most usability testing (A1) used the counseling script at a slightly higher rate. Further studies are needed to examine the independent, and possibly synergistic, associations between these implementation strategies.

Clinician use of the counseling script among patients with moderate- to high-risk alcohol or drug use was low at all clinics. This low adoption may reflect the fact that counseling was recommended but not required and that delivering the script was a complex and relatively time-consuming task (requiring ≥5 minutes).^64,65^ Focus groups conducted in an earlier stage of this study voiced concern that substance use counseling would be too time consuming to include during regular primary care visits.^25^ Further barriers to adoption could have been clinician discomfort and lack of knowledge about alcohol and drug use, which may have made them reluctant to engage patients in conversation about these behaviors.^14,66^

### Limitations

This study has limitations. As an implementation feasibility study, it sought to adapt to the existing conditions and resources of the participating clinics. Clinics were not randomized, so it is not possible to conclude with confidence that the differences we observed are associated with the screening approaches or implementation strategies used rather than other practice characteristics. In addition, clinics had overlapping combinations of implementation strategies, so we are unable to pinpoint whether robust practice facilitation or usability testing has more substantial implications for differential screening outcomes. However, the extent of the differences, particularly in screening rates and the prevalence of unhealthy alcohol use detected via screening, suggests that the screening approach selected by clinics was associated with the outcomes. We had limited ability to measure clinician counseling, and it is possible that clinicians discussed screening results with patients without using the counseling script. Conducting medical record reviews or using natural language processing to analyze clinical notes may have captured this information, but these methods were outside the scope of the study. We were also unable to measure referrals for substance use treatment among patients who received positive screening results for high-risk substance use. Although study clinics varied in size, location, and patient population and included both faculty and resident clinicians, they were all within urban academic health care systems; thus, they are not representative of all primary care practices.

## Conclusions

By examining the outcomes of common approaches to screening for substance use in primary care settings, the findings of this quality improvement study can guide clinics and health care systems that are seeking to implement screening for alcohol and drug use. Interest in screening will likely increase, motivated by US Preventive Services Task Force recommendations,^4,5^ the Healthcare Effectiveness Data and Information Set (HEDIS) measure for alcohol screening and brief interventions,^67^ and increases in substance use associated with the COVID-19 pandemic.^68^ The high screening rate achieved in our study clinics supports the feasibility of EHR-integrated screening for substance use as part of routine primary care. This study also suggests best practices, including the use of self-administered screening tools and the performance of screening at any type of visit. The implementation strategies of robust practice facilitation and usability testing, although more resource intensive, were also associated with greater adoption of screening and counseling.

Many previous studies have reported the challenges of implementing substance use screening and interventions in primary care settings.^26,29,44,46,69,70^ The health care systems participating in the present study have maintained screening and are now adopting our EHR-integrated screening tools systemwide, which highlights the success of their implementation. However, more research is needed regarding beneficial interventions to address moderate-risk drug use during primary care visits, strategies for motivating clinicians to engage patients in discussions of substance use, and the resources required to do so.

## Notes

Supplement. **eFigure.** Frequency of Screening for Alcohol and Drugs and Prevalence of Unhealthy Alcohol and Drug Use by Study Clinic
