# Supplementary material for: Comparison of Methods for Alcohol and Drug Screening in Primary Care Clinics
Source: JAMA Netw Open. 2021 May 20;4(5):e2110721. doi: 10.1001/jamanetworkopen.2021.10721 (PMC8138691; doi:10.1001/jamanetworkopen.2021.10721)
Supplement: Supplement. — eFigure. Frequency of Screening for Alcohol and Drugs and Prevalence of Unhealthy Alcohol and Drug Use by Study Clinic [file jamanetwopen-e2110721-s001.pdf]

Supplementary Online Content

McNeely J, Adam A, Rotrosen J, et al. Comparison of methods for alcohol and drug screening in primary care clinics. *JAMA Netw Open*. 2021;4(5):e2110721. doi:10.1001/jamanetworkopen.2021.10721

eFigure. Frequency of Screening for Alcohol and Drugs and Prevalence of Unhealthy Alcohol and Drug Use by Study Clinic

This supplementary material has been provided by the authors to give readers additional information about their work.

© 2021 McNeely J et al. *JAMA Network Open*.

eFigure. Frequency of Screening for Alcohol and Drugs and Prevalence of Unhealthy Alcohol and Drug Use by Study Clinic

Note: This supplemental figure is a graphic representation of the data included in Table 4 and is submitted for the interest of readers.

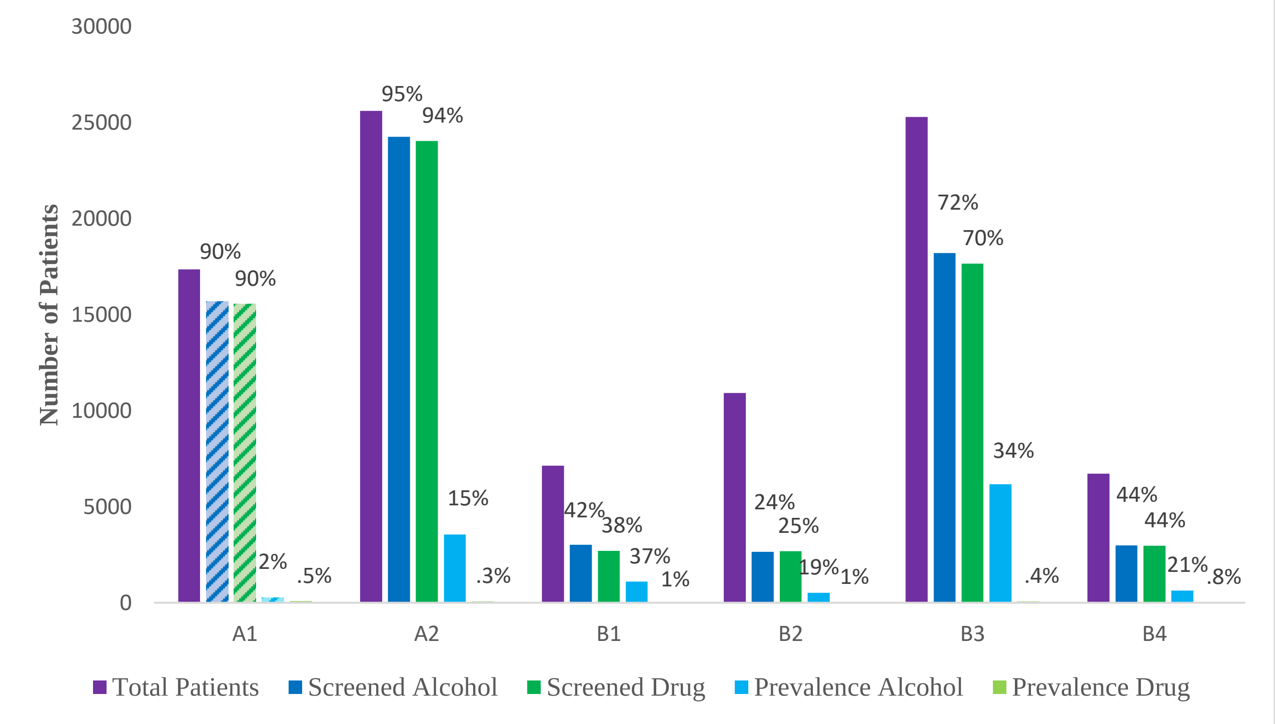

Notes: Diagonal stripes indicate staff administered screening. Data labels on alcohol and drug screening is the percent of total patients screened; data label on prevalence of unhealthy alcohol and drug use is the percent of those screened.

© 2021 McNeely J et al. *JAMA Network Open.*
